# Surface-enhanced Raman spectroscopy liquid biopsy: an emerging technique for the early screening of Alzheimer’s disease

**DOI:** 10.1186/s12967-024-05341-1

**Published:** 2024-06-06

**Authors:** Chuang Qi, Yu Wan, Xiangwei Zhao

**Affiliations:** 1 State Key Laboratory of Digital Medical Engineering, School of Biological Science and Medical Engineering, Southeast University, Nanjing, 211189 China; 2 Institute of Biomaterials and Medical Devices, Southeast University, Suzhou, 215163 China; 3 Southeast University Shenzhen Research Institute, Shenzhen, 518000 China

## To the editor

Alzheimer’s disease (AD) has attracted attention because of the aging society. Medical intervention for mild cognitive impairment (MCI) can substantially reduce AD progression to dementia, thus necessitating the early screening of patients with MCI [1]. Researchers have developed and implemented non-invasive methods to detect AD serum biomarkers, such as single-molecule immunoassay technology (Simoa), mass spectrometry, and chemiluminescence immunoassays, among others. However, the process of operation, assessment costs, and robustness are unsatisfactory in clinical practice. Here, we introduce a novel spectroscopic liquid biopsy method termed surface-enhanced Raman spectroscopy (SERS) for early AD screening.

SERS can provide fingerprint information of molecular vibrations. Therefore, it can be used to distinguish different biomolecules and tissue types. Compared with Simoa and MS, among others, SERS-based assessment is rapid, flexible, and inexpensive, making it a candidate tool for early AD screening as a point-of-care testing (POCT) product. In this letter, we will introduce two impressive POCT products for early AD screening, namely integrated lateral flow immunoassay (LFIA) and microfluidic chip with SERS technology.

## SERS -LFA

LFIA sensors have several advantages, including ease of patterning, flexibility, and low cost. Yuanbao et al. combined SERS and LFIA for the early screening of AD, which significantly improved the detection performance of LFIA. SERS-LFA detected AD-related biomarkers, namely amyloid beta (Aβ) 42, Aβ 40, tau proteins, and the neurofilament light chain with sensitivity at the fg/mL level [2]. External factors in the SERS-LFIA strip affect the SERS signal, supposedly influencing the detection stability and reliability. To eliminate these effects, Xinyu et al. designed a SERS-LFIA strip with a self-calibrating function. Embedding internal standard SERS nanoparticles in the test line of the SERS-LFIA strip reduced SERS signal fluctuations. Additionally, AD-related biomarkers were detected successfully with high sensitivity and specificity of fg/mL level [3].

## SERS microfluidic chip

Microfluidic chip has numerous advantages, including high throughput, low sample requirement, and multifunctional integration. Jianli et al. developed an integrative assay based on SERS and a microfluidic chip for early AD screening. They incorporated polystyrene microspheres with plasmonic gold shells into a microfluidic chip for simultaneously detecting Aβ42 and p-Tau181. The lower limit of detection was 100 fg/ml [4]. The microfluidic platform with SERS technology initiated a novel approach for accurately assessing AD-related biomarkers in human blood samples and elucidated its potential application to clinical practice.

The population of patients with AD is substantial. Considering the accuracy, cost, and sample assessment processing, the clinical application of Simoa, MS, chemiluminescence, and other techniques in the early detection of AD biomarkers in the blood remains challenging. The SERS-LFIA strip and microfluidic SERS chip harbored low cost and accurate performance, despite challenges, such as the standardization of SERS and a larger cohort for clinical evaluation. Xinyuan et al. developed a novel approach termed quantitative technology of digital colloid-enhanced Raman spectroscopy, facilitating the reliable quantitative detection of ultra-low concentration target molecules. This approach lays the foundation for extensive biomarker screening using SERS in early disease stages [5]. In summary, spectroscopic liquid biopsy is an interesting and promising tool for the early screening of AD and showcases substantial application potential.

## References

[1] Hai V, Nguyen S, Mital DS, Knopman G, Caleb Alexander (2024). Cost-effectiveness of Lecanemab for individuals with early-stage Alzheimer Disease. Neurology.

[2] Zhan Y, Fei R, Lu Y, Wan Y, Wu X, Dong J et al. Ultrasensitive detection of multiple Alzheimer’s disease biomarkers by SERS-LFA.Analyst.2022Sep 12;147(18):4124–4131. 10.1039/d2an00717g. 10.1039/d2an00717g 35971961

[3] Xinyu Liu X, Su M, Chen Y, Xie M, Li. Self-calibrating surface-enhanced Raman scattering-lateral flow immunoassay for determination of amyloid-β biomarker of Alzheimer’s disease. Biosens Bioelectron 2024 Feb 1:245115840. 10.1016/j.bios.2023.115840. 10.1016/j.bios.2023.115840 37988777

[4] Jianli S, Wang ZSL, Zhang X, Luo C, Hua J et al. Construction of a microcavity-based microfluidic chip with simultaneous SERS quantification of dual biomarkers for early diagnosis of Alzheimer’s disease. Talanta 2023 Aug 15:261124677. 10.1016/j.talanta.2023.124677. 10.1016/j.talanta.2023.124677 37201340

[5] Xinyuan Bi, Daniel M, Czajkowsky Z, Shao J, Ye. Digital colloid-enhanced Raman spectroscopy by single-molecule counting. Nat 2024 Apr 17. 10.1038/s41586-024-07218-1. 10.1038/s41586-024-07218-1 38632399

